# P-699. Treatment failure and adverse events after amoxicillin-clavulanate versus amoxicillin for acute sinusitis in adults

**DOI:** 10.1093/ofid/ofaf695.911

**Published:** 2026-01-11

**Authors:** Timothy Savage, Anne Mobley Butler, Matthew Kronman, Michael J Durkin, Sushama Kattinakere Sreedhara, Sarah Kabbani, Lauri A Hicks, Krista F Huybrechts

**Affiliations:** Brigham and Women's Hospital / Boston Children's Hospital, Boston, MA; Washington University in St. Louis, St. Louis, MO; Seattle Children's Hospital / University of Washington, Seattle, WA; Washington University School of Medicine, St. Louis, MO; Brigham and Women's Hospital, Boston, Massachusetts; Centers for Disease Control and Prevention, Atlanta, GA; Centers for Disease Control and Prevention, Atlanta, GA; Brigham and Women's Hospital, Boston, Massachusetts

## Abstract

**Background:**

Acute sinusitis ranks among the top conditions leading to antibiotic prescriptions. There is no agreement on the optimal first-line antibiotic; IDSA guidelines provide a weak recommendation supported by low-quality evidence for amoxicillin-clavulanate over amoxicillin. No study has directly compared these antibiotics for treating acute sinusitis in adults.

**Table 1:**
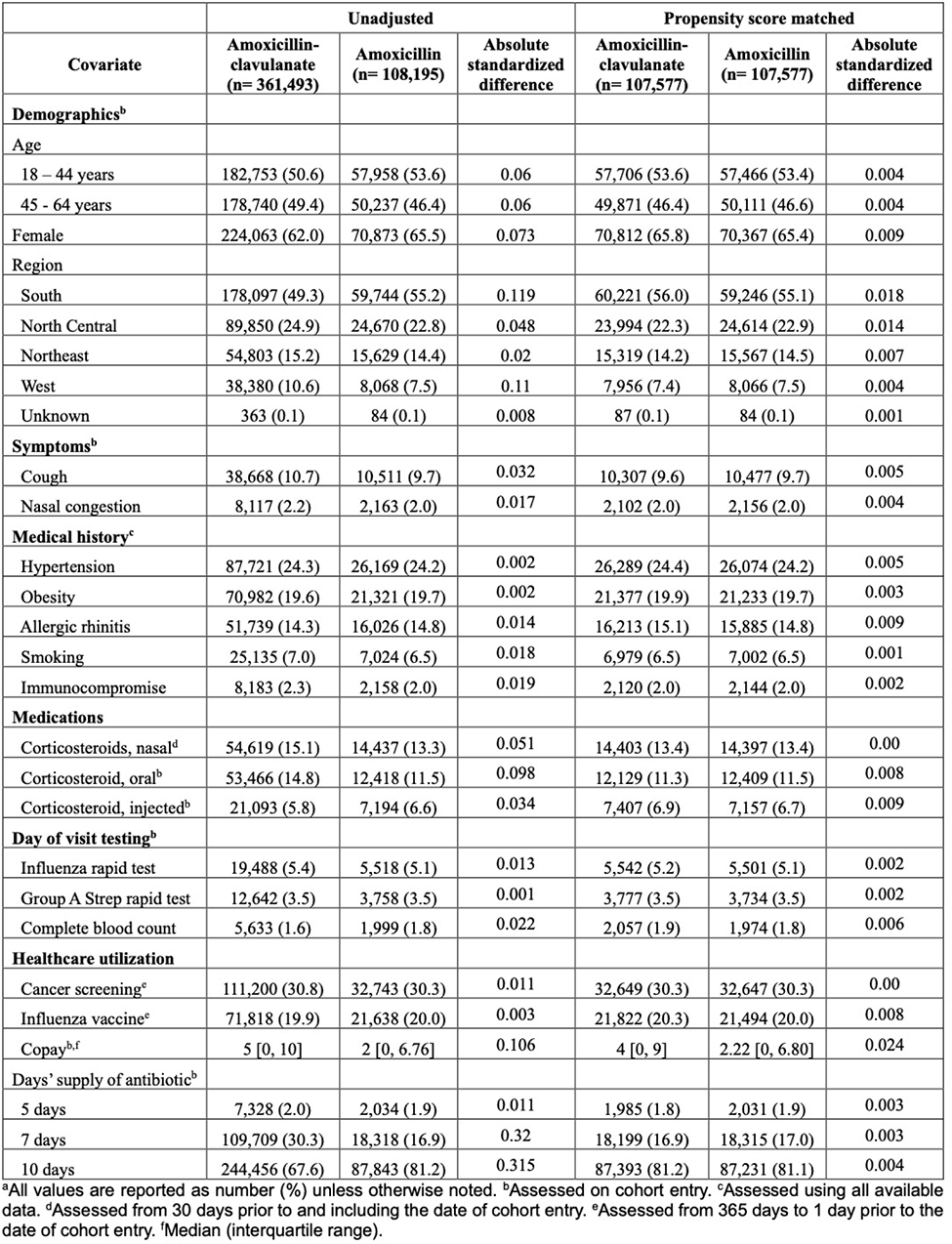
Baseline Demographic and Clinical Characteristics of Adult Sinusitis Patients

**Figure 1:**
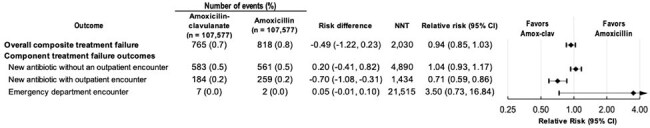
Treatment Failure after Propensity Score Matching

**Methods:**

The cohort included adults 18 to 64 years with an office-based encounter for acute sinusitis (ICD-10: J01.x0) and a same-day dispensation for amoxicillin (875mg BID or 500mg TID) or amoxicillin-clavulanate (875mg-125mg BID) in the MarketScan Commercial Database (2018-2022). Patients with chronic sinus disease or alternative infections were excluded. Treatment failure (subsequent antibiotic dispensation ± an outpatient encounter or an ED/inpatient admission) was evaluated within 14 days after index antibiotic dispensation. Adverse events and negative control outcomes were captured over up to 90 days following treatment initiation. Relative risks (RR) and risk differences (RD) were estimated after 1:1 propensity score matching for confounding control.

**Figure 2:**
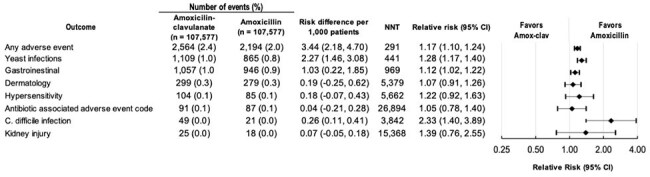
Risk of Adverse Events after Propensity Score Matching

**Figure 3:**
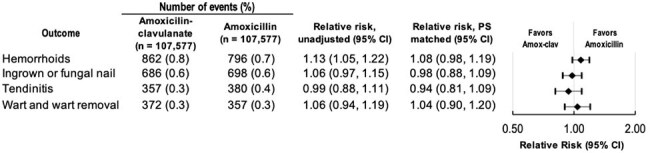
Risk of Negative Control Outcomes before and after Propensity Score Matching

**Results:**

The final cohort included 107,577 propensity score-matched patients per group (Table 1). Treatment failure occurred in 765 (0.8%) patients dispensed amoxicillin-clavulanate and 818 (0.8%) dispensed amoxicillin (RR 0.94 [95% CI, 0.83, 1.03], Figure 1). Amoxicillin-clavulanate was associated with a higher risk of adverse events overall (RR 1.17 [95% CI, 1.10, 1.24]; number needed to harm = 291), as well as several individual adverse events (yeast infection (RR 1.28 [95% CI, 1.17, 1.40]), gastrointestinal symptoms (RR 1.12 [95% CI, 1.02, 1.22]), C. difficile infection (RR 2.33 [95% CI, 1.40, 3.89]) (Figure 2)). Negative control outcomes revealed no difference between groups, suggesting no residual confounding from differential health-seeking behavior (Figure 3).

**Conclusion:**

Among adults with new diagnoses of acute sinusitis treated in the office setting, amoxicillin-clavulanate was associated with no treatment failure benefit but increased risk of harms compared to amoxicillin. When an antibiotic is indicated for adults with sinusitis seen in the office setting, clinicians should consider amoxicillin rather than amoxicillin-clavulanate.

**Disclosures:**

Timothy Savage, MD, MPH, MSc, UCB: Grant/Research Support Krista F. Huybrechts, PhD, MS, Takeda: Grant/Research Support|UCB: Grant/Research Support

